# A Fenofibrate Diet Prevents Paclitaxel-Induced Peripheral Neuropathy in Mice

**DOI:** 10.3390/cancers13010069

**Published:** 2020-12-29

**Authors:** Martial Caillaud, Nipa H. Patel, Wisam Toma, Alyssa White, Danielle Thompson, Jared Mann, Tammy H. Tran, Jane L. Roberts, Justin L. Poklis, John W. Bigbee, Xianjun Fang, David A. Gewirtz, M. Imad Damaj

**Affiliations:** 1 Department of Pharmacology and Toxicology and Translational Research Initiative for Pain and Neuropathy, Medical College of Virginia Campus, Virginia Commonwealth University, Richmond, VA 23284, USA; Wisam.Toma@vcuhealth.org (W.T.); whiteab@mymail.vcu.edu (A.W.); thompsondc3@mymail.vcu.edu (D.T.); mannja@mymail.vcu.edu (J.M.); jane.roberts@vcuhealth.org (J.L.R.); justin.poklis@vcuhealth.org (J.L.P.); 2 Departments of Pharmacology and Toxicology and Medicine and Massey Cancer Center, Virginia Commonwealth University, Massey Cancer Center, Richmond, VA 23284, USA; patelnh3@vcu.edu (N.H.P.); tranth4@mymail.vcu.edu (T.H.T.); david.gewirtz@vcuhealth.org (D.A.G.); 3 Department of Anatomy and Neurobiology, School of Medicine, Virginia Commonwealth University, Richmond, VA 23284, USA; john.bigbee@vcuhealth.org; 4 Department of Biochemistry & Molecular Biology, School of Medicine, Virginia Commonwealth University, Richmond, VA 23284, USA; xianjun.fang@vcuhealth.org

**Keywords:** fenofibrate, PPAR-α, paclitaxel, neuropathic pain, peripheral neuropathy

## Abstract

**Simple Summary:**

Paclitaxel, a drug used in the treatment of malignancies such as lung, ovarian and breast cancer, often produces severe side effects, among which is peripheral neuropathy. This neuropathy involves diffuse or localized pain, notably burning pain, cold and mechanical hyperexcitability. Recently, fenofibrate, a Food and Drug Administration (FDA)-approved drug for the treatment of dyslipidemia, has been shown to reduce the severity of symptoms in other forms of peripheral neuropathy. In the current work, we tested whether fenofibrate could reverse mechanical and cold hypersensitivity and improve motivation and the reduction in nerve conduction in a mouse model of paclitaxel-induced neuropathy. Our behavioral, histological and molecular assessments indicate that fenofibrate prevents the development of paclitaxel-induced neuropathy. Taken together, our studies support the therapeutic potential of fenofibrate in the prevention of paclitaxel-induced neuropathy and suggest the possible repurposing of this drug for this purpose in the clinic.

**Abstract:**

Background: Paclitaxel-induced peripheral neuropathy (PIPN) is a major adverse effect of this chemotherapeutic agent that is used in the treatment of a number of solid malignancies. PIPN leads notably to burning pain, cold and mechanical allodynia. PIPN is thought to be a consequence of alterations of mitochondrial function, hyperexcitability of neurons, nerve fiber loss, oxidative stress and neuroinflammation in dorsal root ganglia (DRG) and spinal cord (SC). Therefore, reducing neuroinflammation could potentially attenuate neuropathy symptoms. Peroxisome proliferator-activated receptor-α (PPAR-α) nuclear receptors that modulate inflammatory responses can be targeted by non-selective agonists, such as fenofibrate, which is used in the treatment of dyslipidemia. Methods: Our studies tested the efficacy of a fenofibrate diet (0.2% and 0.4%) in preventing the development of PIPN. Paclitaxel (8 mg/kg) was administered via 4 intraperitoneal (i.p.) injections in C57BL/6J mice (both male and female). Mechanical and cold hypersensitivity, wheel running activity, sensory nerve action potential (SNAP), sciatic nerve histology, intra-epidermal fibers, as well as the expression of PPAR-α and neuroinflammation were evaluated in DRG and SC. Results: Fenofibrate in the diet partially prevented the development of mechanical hypersensitivity but completely prevented cold hypersensitivity and the decrease in wheel running activity induced by paclitaxel. The reduction in SNAP amplitude induced by paclitaxel was also prevented by fenofibrate. Our results indicate that suppression of paclitaxel-induced pain by fenofibrate involves the regulation of PPAR-α expression through reduction in neuroinflammation. Finally, co-administration of paclitaxel and the active metabolite of fenofibrate (fenofibric acid) did not interfere with the suppression of tumor cell growth or clonogenicity by paclitaxel in ovarian and breast cancer cell lines. Conclusions: Taken together, our results show the therapeutic potential of fenofibrate in the prevention of PIPN development.

## 1. Introduction

Taxanes are microtubule-stabilizing agents that are widely used in the treatment of many solid malignancies such as lung, ovarian and breast cancers [1]. Unfortunately, one of the major side effects of taxanes, such as paclitaxel, is the development of a dose-limiting peripheral and painful neuropathy (PN) that can be irreversible and long-lasting [2,3]. Paclitaxel has been found to cause chemotherapy-induced peripheral neuropathy (CIPN) both acutely and chronically (after 2 years) in 59–78% and 30% of cancer patients, respectively. Painful neuropathy is often severe and its symptoms and severity are dependent on many factors including type of drugs, dose, duration of treatment, prior or concurrent treatment with other drugs and co-existing risk factors [4,5,6]. Symptoms of PN include tingling, numbness, cold and touch allodynia and more diffuse pain. These symptoms significantly interfere with quality of life and may lead to dose reduction in patients who can no longer tolerate the treatment [7]. Thus, the identification of therapeutic targets and the development of treatments that could prevent PN without reducing the anti-tumor effect of paclitaxel has become a major research challenge.

Several studies have shown that activation of the nuclear peroxisome proliferator-activated receptor-α (PPAR-α) can reduce the signs of neuropathy in different models of neuropathic pain [8,9,10]. PPAR-α, like other members of the nuclear receptor superfamily, is activated by ligand binding, which leads to a change in receptor conformation and the formation of heterodimers with retinoid X receptor (RXR), leading to coactivator recruitment and gene transcription. The genes activated by this receptor code for proteins involved in lipid metabolism and inflammation [11]. Thus, it has been shown that the endogenous agonist of PPAR-α palmitoylethanolamide (PEA) exerts analgesic-like properties in rodent models of chronic pain [12,13] and in pilot studies in humans [14]. Significantly, we have recently reported that PEA reversed paclitaxel-induced mechanical allodynia in mice by a PPAR-α mechanism, with no evidence of drug tolerance after chronic administration [15]. In addition, exogenous PPAR-α agonists such as fenofibrate have been shown to exert analgesic and neuroprotective effects in rodent models of chronic neuropathic pain and inflammation as well as in some human studies [8,9,10]. Very recently, activation of PPAR-α by fenofibrate has also been shown to promote sensory nerve fiber regrowth in a model of traumatic nerve injury [16]. Fenofibrate is a Food and Drug Administration (FDA)-approved drug for the treatment of dyslipidemia. It is a prodrug that requires de-esterification in the liver to fenofibric acid, the active metabolite, which is then released into the plasma and transported to the tissues where PPAR-α is expressed [9,17].

Although the therapeutic effect of exogenous PPAR-α agonists such as fenofibrate has previously been studied in a few models of peripheral neuropathy, their potential to prevent paclitaxel-induced peripheral neuropathy (PIPN) has not, to our knowledge, been explored. In this study, we tested the efficacy of dietary fenofibrate (0.2% and 0.4%) administration in preventing the development of PIPN in C57BL/6J male and female mice. To this end, mechanical and cold hypersensitivity, wheel running activity, sensory nerve action potential (SNAP), sciatic nerve histology, intra-epidermal fibers, as well as the expression of PPAR-α mRNA and neuroinflammation were evaluated in dorsal root ganglia (DRG) and spinal cord (SC). Finally, we confirmed the absence of any interaction of fenofibric acid (the fenofibrate main metabolite) with the anti-tumor effect of paclitaxel, which is a crucial consideration if these compounds are to be used as a preventative and/or therapeutic treatment for PIPN in cancer patients.

## 2. Results

### 2.1. Study of Bioavailability and Tolerability of Fenofibrate Diets

**Figure 1:**
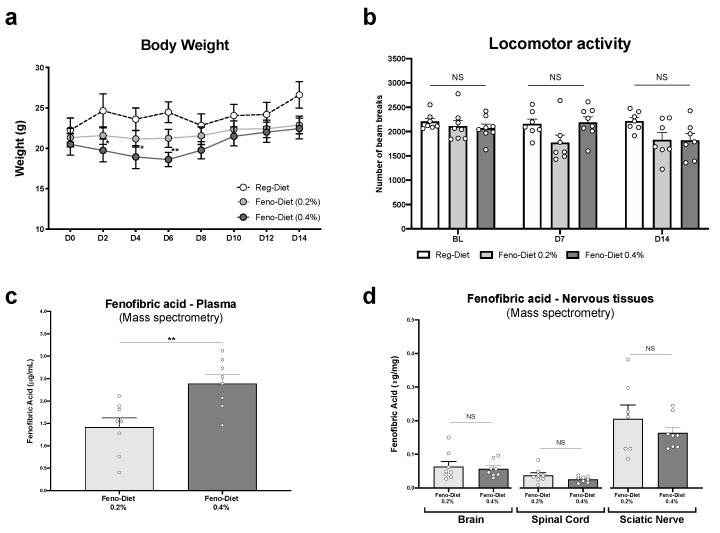
Tolerability of fenofibrate diet and plasma and tissue levels of fenofibrate: (**a**) body weight measurement every other day for two weeks; (**b**) locomotor activity measured at baseline (BL), day 7 (D7) and day 14 (D14) in animals treated with regular, fenofibrate 0.2% and fenofibrate 0.4% diets. (**c**,**d**) Plasma and central and peripheral nervous tissues’ concentration of fenofibric acid, the active metabolite of fenofibrate, measured by mass spectrometry. Values are expressed as mean ± SEM. *n* = 8/group. Results were compared using two-way ANOVA (**a**), one-way ANOVA (**b**) with post-hoc Tukey’s test and *t*-test (**c**,**d**) (*: *p* < 0.05, **: *p* < 0.01 vs. vehicle). No sex differences were observed (data not shown).

An initial study was conducted on a cohort of mice not treated with paclitaxel to determine whether the 0.2% and 0.4% fenofibrate doses in the diet over a period of two weeks would be well tolerated by the animals (*n* = 8 animals/group) (Figure 1). Mice were monitored daily to assess their general health status, and no signs of deterioration of their health were observed. No weight loss was observed in the mice treated with the 0.2% fenofibrate diet. However, at day 2 (D2) (*p* < 0.05), D4 (*p* < 0.05) and D6 (*p* < 0.01), a significant but modest decrease in body weight was observed in the group treated with the 0.4% fenofibrate diet compared to the control group. The mice then regained weight rapidly (D8) and weight was stabilized between D10 and D14 (Figure 1a).

Due to rare cases of reports of muscle side effects (muscular weakness, muscle pain and cramps), primarily when fenofibrate is administered in combination with other drugs such as statins [18], the locomotor activity of mice given the fenofibrate diet was assessed, again in the absence of paclitaxel treatment. Our data show that the locomotor activity of mice treated with the 0.2% and 0.4% fenofibrate diets did not significatively change at D7 and D14 for both regimens compared to the control group (Figure 1b).

Finally, plasma and nervous tissues’ concentration of fenofibric acid, the active metabolite of fenofibrate, was measured by mass spectrometry (MS) at day 15 of treatment with the 0.2% and 0.4% fenofibrate diets (Figure 1c,d). It is important to note that only fenofibric acid was detected in the samples and that fenofibrate was not detected. Statistical analysis (*t*-test) of our data shows a significantly higher plasma concentration in the group of mice treated with the 0.4% fenofibrate diet (*p* < 0.01) than the 0.2% diet. However, no significant difference in the concentration of fenofibric acid was observed in the brain, spinal cord and sciatic nerve between the two diets.

### 2.2. Fenofibrate Diets Reduce Signs of Paclitaxel-Induced Peripheral Neuropathy

We next investigated whether exposure to the fenofibrate diet could prevent various PIPN behavioral, physiological and morphological measures (*n* = 12 animals/group). In order to determine the impact of paclitaxel and fenofibrate diets on voluntary wheel running activity, the animals performed the wheel running test on day 7 (this day having been chosen according to our previous publication [19]). The overall ANOVA showed a significant difference between the groups (*p* = 0.026). Specifically, no significant difference was observed between the three vehicle groups treated with the regular diet, fenofibrate (Feno) 0.2% or 0.4%. However, a significant decrease in distance travelled was observed in the paclitaxel-regular diet group compared to the control group (*p* < 0.05). In contrast, the two groups treated with paclitaxel and fenofibrate diets of 0.2% and 0.4% showed no difference from the control group. These results suggest that fenofibrate can prevent the decrease in wheel running activity produced by paclitaxel.

Mechanical sensitivity was then measured at different times (BL, D0, D7 D14 and D21) by the Von Frey filament test. The overall ANOVA showed an interaction between time and treatment factors (F (20, 264) = 8.628; *p* < 0.0001). More precisely, no difference in mechanical sensitivity was observed between the three vehicle groups at the different measurement points (BL to D21). However, a significant decrease in the paw withdrawal threshold was observed in the paclitaxel-treated animals at D7 (*p* < 0.001), D14 (*p* < 0.001) and D21 (*p* < 0.001), reflecting paclitaxel-induced mechanical hypersensitivity. Treatment with the fenofibrate diet at 0.2% partially prevented this hypersensitivity at D7 and D14. The protection induced by the treatment was lost at D21. Nevertheless, treatment with the fenofibrate diet at 0.4% completely prevented mechanical hypersensitivity induced by paclitaxel to D7 and partially to D14 and D21. Taken together, these results demonstrate the ability of fenofibrate to prevent the development of paclitaxel-induced mechanical hypersensitivity.

**Figure 2:**
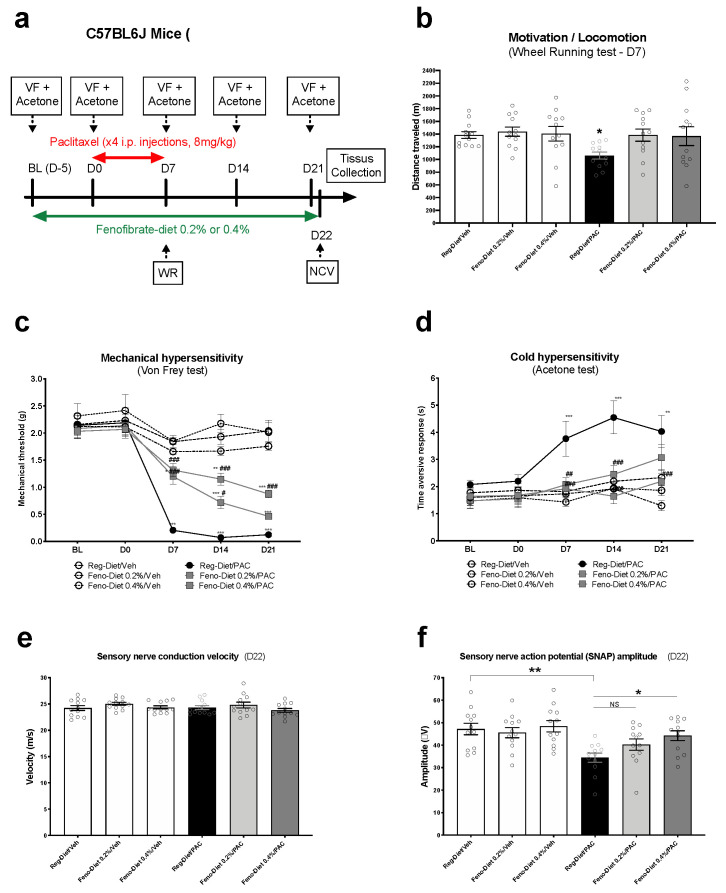
Prevention of paclitaxel-induced peripheral neuropathy (PIPN) signs with fenofibrate diet treatment: Mice were treated with 0.2% or 0.4% fenofibrate diets for 4 weeks (before, during and after paclitaxel) (**a**). Motivation and locomotion were evaluated by the wheel running test at D7 (**b**). Mechanical and cold (**c**,**d**) hypersensitivity were tested at BL, D0, D7, D14 and D21 with the Von Frey and acetone tests, respectively. Sensory nerve conduction velocity was measured at D22 (**e**). Sensory nerve compound action potential amplitude was measured at D22 (**f**). Values are expressed as mean ± SEM. *N* = 12/group. Results were compared using two-way ANOVA (**c**,**d**) and one-way ANOVA (**b**,**e**,**f**) and post-hoc Tukey’s test (*: *p* < 0.05, **: *p* < 0.01 and ***: *p* < 0.001 vs. vehicle; (#: *p* < 0.05, ##: *p* < 0.01 and ###: *p* < 0.001 vs. paclitaxel group). BL = Baseline; D = day; Feno = Fenofibrate; Reg = Regular; PAC = paclitaxel; Veh = Vehicle. No sex differences were observed (data not shown).

For cold hypersensitivity, the two-way ANOVA showed an interaction between the two factors of treatment and time (Figure 2d: [F (4, 263) = 13.29; *p* < 0.0001]). More specifically, a significant increase in the time aversive response was observed at D7 to D21 (*p* < 0.001 and 0.01), reflecting cold hypersensitivity induced by paclitaxel (Figure 2d). Fenofibrate diets at 0.2% and 0.4% completely prevented the development of cold hypersensitivity at D7 and D14, while only the dose of 0.4% was effective to D21 (Figure 2d). No significant difference was observed between the two fenofibrate diets at 0.2% and 0.4 and the control groups (Figure 2d). Taken together, these results demonstrate the ability of fenofibrate to also prevent the development of paclitaxel-induced cold hypersensitivity.

Finally, our electrophysiological data show a decrease in sensory nerve compound action potential (SNCAP) amplitude (*p* < 0.01) in the paclitaxel-treated group (Figure 2f: one-way ANOVA; F = 4.834; *p* < 0.001). No significant difference was observed between the control group and 0.2% and 0.4% fenofibrate diet-treated groups (Figure 2f). However, a significant difference was evident between the fenofibrate diet 0.4% + paclitaxel group and the paclitaxel-treated group (*p* < 0.05); Figure 2f. Finally, no significant differences were observed in sensory nerve conduction velocity between any of the groups (Figure 2e). Collectively, these results suggest that fenofibrate prevents the decrease in sensory nerve conduction amplitude induced by paclitaxel.

### 2.3. Fenofibrate Diet Prevents the Decrease in Density of Intra-Epidermal Nerve Fibers

**Figure 3:**
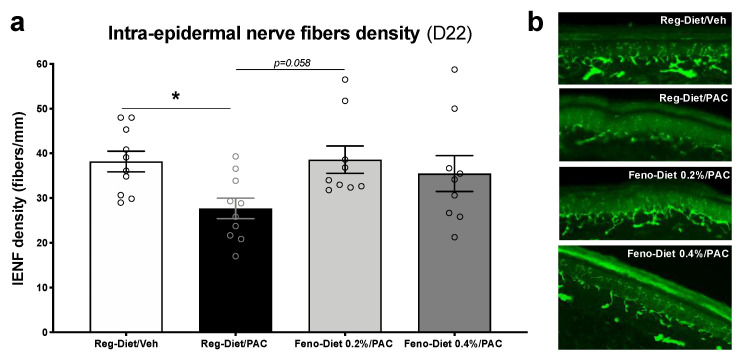
Fenofibrate diet mitigates the reduction in intra-epidermal nerve fiber (IENF) density at 21 days post-paclitaxel injection. (**a**) Quantification of IENF density in hind paw skin at day 21 post-treatment. (**b**) Immunostained sections of Regular-diet/Veh, Regular-diet/Paclitaxel, Fenofibrate diet 0.2%/Paclitaxel and Fenofibrate diet 0.4%/Paclitaxel hind paw skin showing the lENFs. Values are expressed as mean ± SEM. *n* = 10/group. Results were compared using two-way or one-way ANOVA with post-hoc Tukey’s test and *t*-test (*: *p* < 0.05 vs. vehicle). D = day; Feno = Fenofibrate; Reg = Regular; PAC = paclitaxel; Veh = Vehicle. No sex differences were observed (data not shown).

Changes in the density of small nerve fibers innervating the epidermis are an important feature of the CIPN [20]. Thus, in order to evaluate the impact of paclitaxel and fenofibrate diet treatment on intra-epidermal nerve fibers (IENFs), an analysis of IENF density was performed using immunohistochemistry. At 21 days post-paclitaxel injection, mice treated with paclitaxel (Reg-diet/PAC) demonstrated significant reductions in the density of IENFs in comparison to the vehicle group (Reg-Diet/Veh) (*p* < 0.05; Figure 3a). No significant differences were observed between the vehicle group (Reg-Diet/Veh) and both fenofibrate-treated groups (Feno 0.2%-Diet/PAC and Fenofibrate 0.4%-Diet/PAC). However, an increasing trend of IENF density was observed in the Fenofibrate 0.2%-Diet/PAC group in comparison to the Reg-diet/PAC group (*p* = 0.058) (Figure 3). These data suggest that the fenofibrate treatment prevents the loss of IENFs from the paclitaxel treatment.

### 2.4. Fenofibrate Diet Reduces Mitochondria Damage

**Figure 4:**
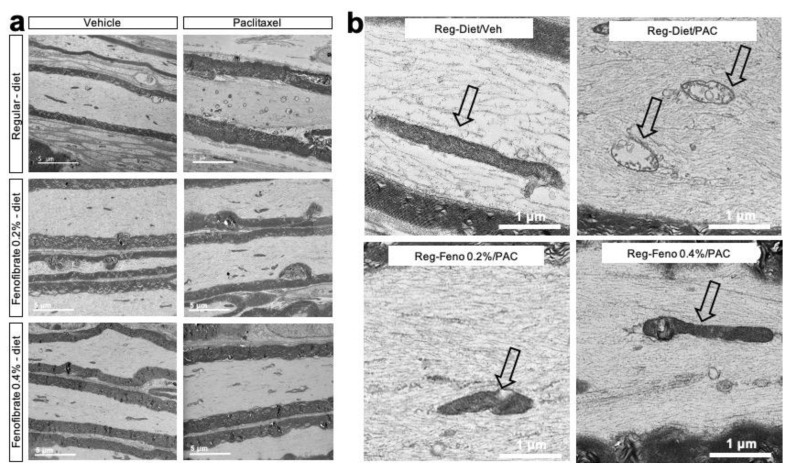
Morphological features of sciatic nerves: Transmission electron microscopy pictures of sciatic nerve longitudinal sections after different treatments (**a**,**b**). Magnification on mitochondria (black arrow) showing mitochondrial ultrastructure in the control group (Vehicle), the paclitaxel-treated group (Paclitaxel), the paclitaxel + fenofibrate 0.2%-treated group and the paclitaxel + fenofibrate 0.4%-treated group (**b**). Feno = Fenofibrate; Reg = Regular; PAC = paclitaxel; Veh = Vehicle.

Since the neurotoxicity of paclitaxel is known to damage intra-axonal mitochondria, a qualitative morphological evaluation by electron microscopy was carried out on longitudinal sections of the sciatic nerve [21] (Figure 4). Our microscopic observation of the nerves revealed that the mitochondria of the control group (Reg-diet/Veh) are, for the most part, elongated in appearance, although some are oval or spherical. The double membrane and the mitochondrial cristae are easily identifiable, reflecting the healthy appearance of the mitochondria. These observations are the same in both myelinated and non-myelinated axons.

In contrast, for the group of animals treated with paclitaxel (Reg-diet/PAC), there was clear evidence of mitochondria pathology that is consistent with paclitaxel toxicity. Thus, we observed swelling of the mitochondria and reduced numbers of elongated mitochondria, the presence of intra-mitochondrial vacuoles, disruption of the mitochondrial membranes and the presence of mitochondrial clustering. However, there were normal-appearing, elongated mitochondria with minimal disruption. This is true for both myelinated and non-myelinated axons. At this time (day 21), there did not appear to be any significant alterations of the cytoskeleton (Figure 4).

Treatment with fenofibrate at both doses reduced the mitochondrial pathology. In the fenofibrate 0.2% group, there were more normal-appearing mitochondria than in the paclitaxel group. In the group of animals treated with fenofibrate 0.4% (Feno-diet 0.4%/PAC), there was a tendency for more elongated mitochondria than in the group treated with fenofibrate 0.2% (Feno-diet 0.2%/PAC) and the group treated with paclitaxel (Reg-diet/PAC). In addition, no mitochondria clustering was observed in either fenofibrate-treated group. However, some mitochondria still showed pathology, including intra-mitochondrial vacuole. These observations reveal an improvement in the ultrastructure of the mitochondria with fenofibrate treatment compared to the group of animals treated with paclitaxel, with a more pronounced effect at the 0.4% fenofibrate dose (Figure 4).

### 2.5. Fenofibrate Diet Increases PPAR-α mRNA Expression in DRG and Spinal Cord

**Figure 5:**
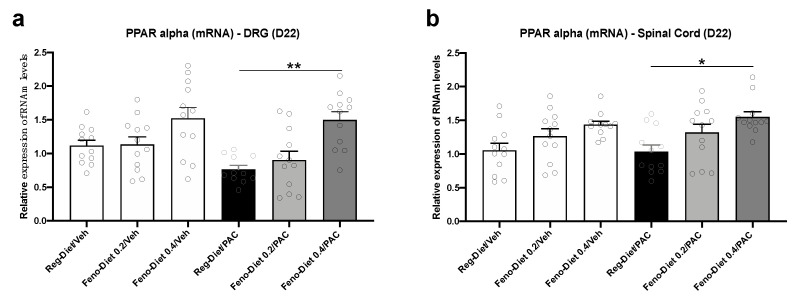
Peroxisome proliferator-activated receptor-α (PPAR-α) mRNA expression in dorsal root ganglia (DRG) and spinal cord: PPAR-α mRNA expression was measured at day 22 after paclitaxel injection in DRG (**a**) and spinal cord (**b**). Values are expressed as mean ± SEM. *n* = 12/group. Results were compared using one-way ANOVA and post-hoc Tukey’s test (*: *p* < 0.05, **: *p* < 0.01). D = day; Feno = Fenofibrate; Reg = Regular; PAC = paclitaxel; Veh = Vehicle. No sex differences were observed (data not shown).

In order to evaluate the impact of fenofibrate diet treatment on the expression of its target receptor, PPAR-α, and its regulation by paclitaxel, the level of PPAR-α mRNA expression was quantified in DRG and spinal cord. Our results show no significant difference in the expression of PPAR-α mRNA in the DRG of animals treated with paclitaxel (Reg-Diet/PAC). However, a decreasing trend that was observed by paclitaxel treatment (Figure 5a) was reversed by fenofibrate. Indeed, a significant increase in the expression of PPAR-α was observed in the DRG in the Feno-diet 0.4%/PAC group in comparison to the Reg-Diet/PAC group (*p* < 0.01) (Figure 5a).

No changes in PPAR-α mRNA expression were observed in the spinal cord of Reg-Diet/PAC mice in comparison to Reg-diet/Veh group (Figure 5b). However, the 0.4% fenofibrate diet (Feno-diet 0.4%/PAC) also significantly increased the expression of PPAR-α mRNA in the spinal cord of mice (*p* < 0.05) (Figure 5b).

### 2.6. Fenofibrate Diet Reduces Inflammatory Markers in DRG and Spinal Cord

**Figure 6:**
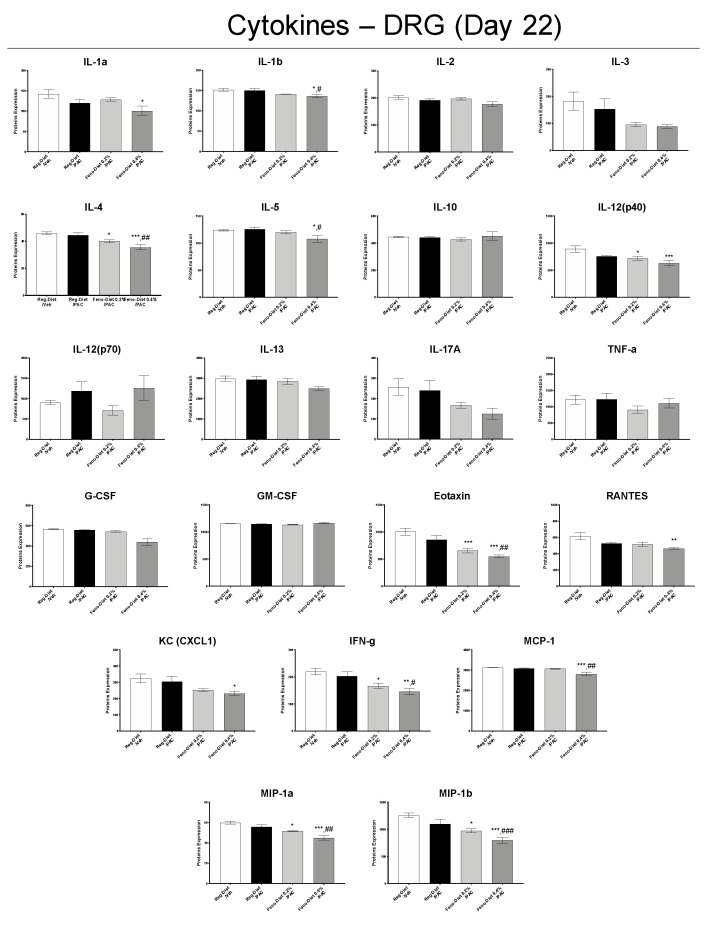
Measurement of inflammation markers in DRG 22 days after injection of paclitaxel with or without 0.2% or 0.4% fenofibrate diet. Values are expressed as mean ± SEM. *n* = 8/group. Results were compared using one-way ANOVA and post-hoc Tukey’s test (*: *p* < 0.05, **: *p* < 0.01 and ***: *p* < 0.001 vs. vehicle group; #: *p* < 0.05, ##: *p* < 0.01 and ###: *p* < 0.001 vs. paclitaxel group). D = day; Feno = Fenofibrate; Reg = Regular; PAC = paclitaxel; Veh = Vehicle. No sex differences were observed (data not shown).

**Figure 7:**
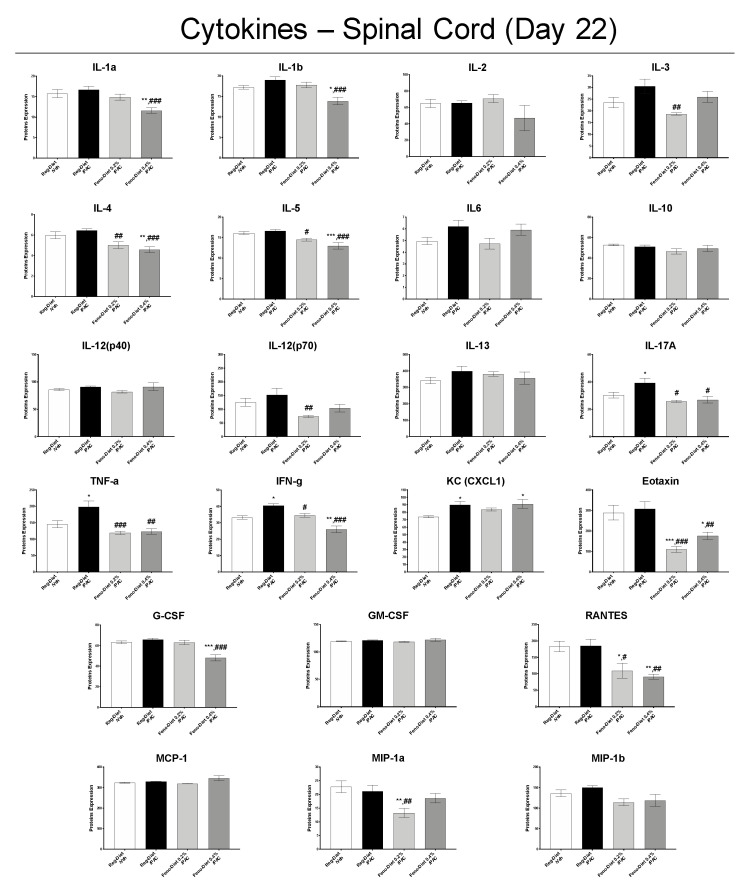
Measurement of inflammation markers in spinal cord 22 days after injection of paclitaxel with or without 0.2% or 0.4% fenofibrate diet. Values are expressed as mean ± SEM. *n* = 8/group. Results were compared using one-way ANOVA and post-hoc Tukey’s test (*: *p* < 0.05, **: *p* < 0.01 and ***: *p* < 0.001 vs. vehicle group; #: *p* < 0.05, ##: *p* < 0.01 and ###: *p* < 0.001 vs. paclitaxel group). D = day; Feno = Fenofibrate; Reg = Regular; PAC = paclitaxel; Veh = Vehicle. No sex differences were observed (data not shown).

In order to determine the effect of paclitaxel and fenofibrate treatment on inflammation, an analysis of the expression profiles of the main inflammatory markers was performed in the DRG (Figure 6) and spinal cords (Figure 7) by Multiplex. The data showed no significant change in the expressions of the different cytokines in the DRG between the control group (Reg-Diet/Veh) and the paclitaxel-treated group (Reg-Diet/PAC) at day 22 (Figure 6). However, treatment with 0.2% fenofibrate (Feno-Diet 0.2%/PAC), significantly decreased the expression of the cytokines Interleukin (IL)-4, IL-12(p40), Eotaxin, interferon (IFN)-γ, macrophage inflammatory proteins (MIP)-1a and MIP-1b compared to the control group (Reg-Diet/Veh) (Figure 6). Treatment with 0.4% fenofibrate (Feno-Diet 0.4%/PAC) significantly reduced levels of the cytokines IL-1β, IL-4, IL-5, IL-12(p40), Eotaxin, RANTES, keratinocytes-derived chemokine (KC), MCP-1, IFN-γ, MIP-1a and MIP-1b compared to the control group (Reg-Diet/Veh). Interestingly, treatment with 0.4% fenofibrate also significantly decreased the expression of the cytokines IL-1β, IL-4, IL-5, Eotaxin, KC, MCP-1, IFN-γ, MIP-1a and MIP-1b compared to the paclitaxel-treated group (Reg-Diet/PAC) (Figure 6).

Analysis of inflammatory markers in the spinal cord at D22 showed a significant increase in the pro-inflammatory cytokines IL-17A, TNF-α, IFN-γ and KC in the paclitaxel-treated group compared to the control group (Figure 7). A trend of increase was also observed for IL-1β, IL-3 and IL-6 (Figure 7). These increases in the pro-inflammatory cytokines IL-17A, TNF-α and IFN-γ were prevented by both doses of fenofibrate (0.2% and 0.4%). In addition, both doses of fenofibrate significantly reduced the levels of IL-4, IL-5, Eotaxin and RANTES compared to the paclitaxel-treated group, and the 0.2% fenofibrate treatment also decreased the expression of IL-3, IL-12(p40) and MIP-1a (Figure 7).

### 2.7. Fenofibric Acid Did Not Alter Paclitaxel-Induced Cytotoxicity in Breast and Ovarian Cancer Cells

**Figure 8:**
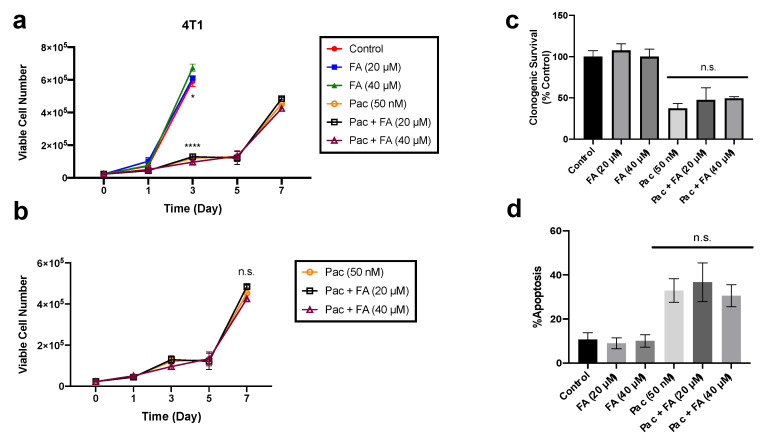
Fenofibric acid did not interfere with paclitaxel-induced cytotoxicity in breast tumor cells. (**a**,**b**) Cell viability: 4T1 cells were exposed to fenofibric acid (FA, 20 or 40 μM), paclitaxel (Pac, 50 nM) or a combination of both for 24 h. The number of viable cells was monitored over 7 days via Trypan blue exclusion. Cell viability over time was graphed for all conditions (**a**) and paclitaxel-treated conditions (**b**) (*n* = 3). (**c**) Clonogenic survival cells were exposed to fenofibric acid (20 or 40 μM), paclitaxel (50 nM) or a combination for 24 h. Cells were allowed to grow for ~11 days with fresh medium, and colonies were stained and counted (*n* = 3). (**d**) Extent of apoptosis: cells were stained with annexin-V and propidium iodide (PI) and quantified using flow cytometry to assess the extent of apoptotic cells following 48 h treatment with fenofibric acid (20 or 40 μM), paclitaxel (50 nM) or a combination for 24 h (*n* = 3). Results are from three independent experiments. * *p* < 0.05, **** *p* < 0.0001 compared to controls. Not significant (n.s.) compared to paclitaxel treated groups.

Given the potential therapeutic promise of fenofibrate and its metabolite fenofibric acid, for the treatment of chemotherapy-induced peripheral neuropathy (CIPN), we evaluated the influence of fenofibric acid on sensitivity to paclitaxel in 4T1 breast cancer cells and SKOV-3 and CAOV3 ovarian cancer cells. The 4T1 cells were exposed to fenofibric acid (20 or 40 μM) or 50 nM paclitaxel alone or in combination with fenofibric acid for 24 h. Cell viability was examined over time to assess the effects of fenofibric acid on paclitaxel-induced growth inhibition (Figure 8a,b). Paclitaxel significantly reduced the viability of 4T1 cells when compared to controls (Figure 8a); administration of fenofibric acid at either the 20 or 40 μM concentration did not interfere with paclitaxel-induced growth inhibition (Figure 8b). In support of these observations, paclitaxel treatment also significantly reduced clonogenic survival of the 4T1 breast cancer cells; this suppression of tumor cell survival by paclitaxel was not compromised by the exposure to fenofibric acid (Figure 8c). The effects of fenofibric acid on the extent of paclitaxel-induced cell death were also examined. Treatment with paclitaxel induced significant apoptotic cell death when compared to untreated controls; as with the growth inhibition and cell survival studies, exposure to fenofibric acid did not interfere with paclitaxel-induced apoptotic cell death (Figure 8d).

**Figure 9:**
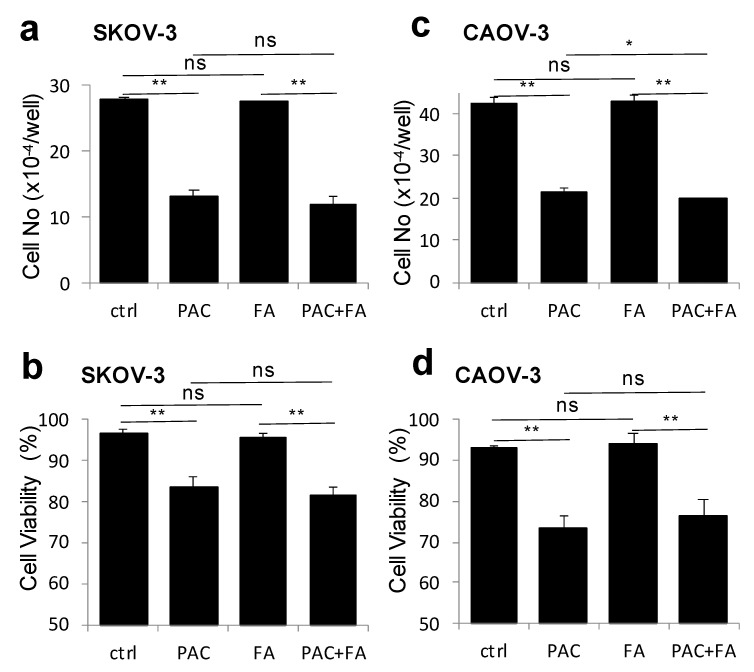
Fenofibric acid did not interfere with paclitaxel-induced cytotoxicity in ovarian tumor cells. SKOV-3 and CAOV3 cells in 12-well plates were treated with paclitaxel (PAC, 7.5 nM in SKOV-3 and 25 nM in CAOV-3), fenofibric acid (FA, 40 mM) or their combination (PAC+FA) for 2 days. The cells were trypsinized and cell numbers/well were quantified with a Coulter counter (**a**,**c**). The viabilities of treated cells including both adherent and non-adherent cells were determined with Trypan blue exclusion (**b**,**d**). The results are presented as mean + SD of triplicate assays. (*: *p* < 0.05, **: *p* < 0.01. Not significant (ns) compared to paclitaxel treated groups.)

SKOV-3 and CAOV3 cells were respectively exposed to paclitaxel 7.5 and 25 nM and to fenofibric acid (40 μM), either alone or in combination for 48 h. Paclitaxel significantly reduced the number of cells and the viability of SKOV-3 and CAOV3 cells when compared to controls (Figure 9a–d). The exposure to fenofibric acid did not interfere with paclitaxel-induced cell death in SKOV-3 and CAOV3 cells (Figure 9a–d).

## 3. Discussion

Paclitaxel-induced peripheral neuropathy is considered a major side effect endured by patients receiving chemotherapy. Unfortunately, these symptoms (burning pain, cold and mechanical allodynia, etc.) can cause patients to choose to reduce the dose of chemotherapy, change the chemotherapeutic agent or discontinue the treatment [3,20]. To date, we only have unsatisfactory symptomatic treatments available to reduce pain, such as antidepressants, duloxetine and morphine [22]. Unfortunately, no treatment is currently available for mitigation or even prevention of the neuropathy induced by paclitaxel and other drugs such as cisplatin, vincristine or bortezomib. Our data from the current study demonstrate that repurposing the currently approved FDA drug fenofibrate, a PPAR-α agonist which is used to treat dyslipidemia, might be an effective therapy to prevent PIPN symptoms. Here, we show that mice fed with a fenofibrate diet demonstrate a partial prevention of mechanical hypersensitivity and full protection against the cold hypersensitivity induced by paclitaxel. Furthermore, fenofibrate treatment restored the decrease in motivation and locomotion in paclitaxel-treated mice. Additionally, the reduction in both amplitude of sensory nerve action potential and intraepidermal nerve fiber density was reversed when a fenofibrate-containing diet was administered to paclitaxel-treated mice. Moreover, treatment with fenofibrate reduced mitochondrial damage and neuroinflammation induced by paclitaxel and increased PPAR-α expression levels in the DRG and spinal cords. Finally, fenofibrate did not interfere with the anti-tumor effects of paclitaxel against cancer cell lines.

Our initial studies on naïve adult male and female C57 BL/6J mice (not treated with paclitaxel) fed a fenofibrate diet with a concentration of 0.4% resulted in only a slight and transient reduction in mouse body weight on days 2, 4 and 6. This is not surprising, since several studies reported that fenofibrate led to a reduction in the body lipid storage and accumulation [23,24,25]. No changes were observed in the general locomotor activity of the mice, suggesting that fenofibrate does not induce any motor depression or muscle pain and weakness at the concentrations used in the current study. Furthermore, we show that dietary fenofibrate increased the plasma levels of fenofibric acid, the primary active metabolite of fenofibrate, in a dose-related manner in mice. The plasma levels of fenofibric acid are consistent with those observed in humans after administration of a 200 mg tablet [26]. In addition, we show that fenofibric acid is well distributed in nervous tissues, including the brain, spinal cord and the sciatic nerve. These initial results allowed us to test dietary fenofibrate (at concentrations of 0.2% and 0.4%) in our previously published animal model of paclitaxel-induced peripheral neuropathy [27].

We recently reported that paclitaxel at a dose of 8 mg/kg, i.p., leads to a reduction in motivation/locomotion in mice at day 7 after the first injection of paclitaxel, measured with the wheel running test [19]. Our current results show that 0.2% and 0.4% fenofibrate dietary supplementations restored the decreased motivation/locomotion observed in mice treated with paclitaxel. In addition, weekly testing of mice treated with paclitaxel revealed significant development of both mechanical and cold hypersensitivity. Mice treated with paclitaxel and fed the fenofibrate diet at concentrations of 0.2% and 0.4% displayed partial prevention of mechanical hypersensitivity and full protection against cold hypersensitivity in a dose-related manner. Additionally, paclitaxel produced a significant reduction in the sensory nerve action potential and intraepidermal nerve fibers. These electrophysiological–histological changes are in accordance with several reports that demonstrate that paclitaxel can induce both sensory and morphological changes in rodents and humans [5,27,28,29]. Nonetheless, in paclitaxel-treated mice with dietary fenofibrate supplementation, the reduction in sensory nerve action potential and nerve fibers in the skin was prevented.

It is commonly accepted that a decrease in the amplitude of the nerve action potential is correlated with a decrease in the number of nerve fibers (axons) and that the nerve conduction velocity is correlated with the myelin thickness [30,31]. The absence of nerve conduction reduction reflects the fact that there is no demyelination of nerve fibers in this PIPN model. However, the decrease in the amplitude of the sensory nerve action potential reflects a paclitaxel-induced decrease in the number of sensory nerve fibers that was prevented by the fenofibrate treatment. Thus, the protective effect of fenofibrate treatment observed here could be explained by a reduction in nerve fiber degeneration and/or stimulation of nerve fiber regrowth. Recently, fenofibrate has been shown to promote the sensory nerve fiber response in a model of traumatic nerve injury by activation of PPAR-α receptors [16]. The results of this study suggest that fenofibrate can be used to activate PPAR-α receptors in the satellite glial cells of DRG and improve the regeneration and growth of sensory axons following injury to dorsally projecting nerves in the spinal cord or repair of peripheral nerves [16].

Fenofibrate may also protect nerve fibers from degeneration by its neuroprotective effect at the level of the mitochondria. Indeed, one of the consequences of the neurotoxicity of paclitaxel is a morphological and functional alteration of the mitochondria of nerve fibers [3,32,33]. The induced damage to the mitochondria is due, in part, to the binding of paclitaxel to the mitochondrial β-tubulin, which can produce a release of Ca^2+^ from the mitochondria and a deregulated intracellular homeostasis of Ca^2+^ [21,34]. Paclitaxel also contributes to a significant increase in the release of reactive oxygen species (ROS), leading to a reduction in mitochondrial membrane potential [21]. Thus, we observe the presence of ovoid, swollen mitochondria with intra-mitochondrial vacuoles, even 22 days after the first injection of paclitaxel. Interestingly, the fenofibrate treatment prevented and limited the damage to the mitochondria of the nerve fibers in a dose-related manner. These results are consistent with a recent study conducted in a cisplatin-induced ototoxicity model [35]. In this study, treatment with fenofibrate reduced the ROS production and suppression of the nuclear factor-kappa B (NF-κB) pathway and nicotinamide adenine dinucleotide phosphate (NADPH) oxidase-3 (NOX3) and NOX4 expression by activation of PPAR-α and poly ADP-ribose polymerase (PGC-1α). Thus, treatment with fenofibrate made it possible to save peroxisomal and mitochondrial functions [35]. Moreover, it has been shown in a chronic neuropathic pain model (chronic constriction injury - CCI) that the use of PPAR-α agonists reduces tissue damage (number of nerve fibers, myelin sheath, etc.). This was attributed to the ability of PPAR-α receptors to reduce cyclooxygenase-2 (COX-2) levels and prevent macrophage infiltration [36]. Thus, this limitation of macrophage infiltration by PPAR-α would also contribute to limiting inflammation and neuropathic pain.

The protective effect of fenofibrate observed in this study may also be mediated by its direct PPAR-α-dependent anti-inflammatory action, as fenofibrate is a PPAR-α receptor agonist. Indeed, we observed a significant increase in PPAR-α mRNA expression in DRG and spinal cord with the 0.4% fenofibrate diet, as well as a decrease in inflammatory markers directly mediated by the PPAR-α-dependent pathway. PPAR-α are nuclear receptors leading to numerous genes regulating, notably, proteins involved in lipid metabolism and inflammation [11]. PPAR-α regulates systemic inflammation by inducing the expression of anti-inflammatory proteins such as IκB-α and repressing the Nf-κB pathway and the expression of proinflammatory proteins such as tumor necrosis factor (TNF)-α, IL-1 and IL-6, while limiting the recruitment of immune cells [11,37]. This anti-inflammatory effect of PPAR-α is all the more interesting since paclitaxel-induced inflammation has been widely described in the literature [38,39,40]. Our results showed no difference in the expression of pro-inflammatory cytokines in DRG. However, a significant increase in IL-17A, TNF-α, IFN-γ and KC, as well as a trend for IL-1β, IL-3 and IL-6, was observed in the spinal cord 21 days after the first administration of paclitaxel. This is not surprising as it has been reported that inflammation in the DRG was greatest in the early stages of neuropathy (day 7–10) and then present in the spinal cord at the later stages, maintained by glial activation [3,38,39,40]. Both doses of fenofibrate reduced the expression of these pro-inflammatory cytokines as well as other markers of inflammation such as IL-4, IL-5, Eotaxin, RANTES, IL-12(p40) and MIP-1a. Some of these inflammatory markers, such as TNF-α, IFN-γ, IL-1, IL-6 and MIP-1a, have been reported to be downregulated by PPAR-α activation [11]. These results, therefore, seem to confirm the anti-inflammatory effect of PPAR-dependent fenofibrate. Thus, this decrease in inflammation may play a role in the reduction in hypersensitivity observed in functional tests. Collectively, fenofibrate, through the activation of PPAR-α, represents a promising treatment in the prevention of PIPN through its antioxidant, anti-inflammatory and axonal regrowth-stimulating action.

Finally, prior to considering the translation of these agents to the clinic for the treatment of PIPN, it was important to evaluate fenofibric acid in pre-clinical cancer models, both alone and in combination with paclitaxel. We tested fenofibric acid and not fenofibrate in the tumor cell lines because our bioavailability experiments indicated only the presence of fenofibric acid in plasma and nerve tissue and no traces of fenofibrate. Our results in ovarian and breast cancer cell lines confirmed that fenofibric acid does not promote tumor cell growth or attenuate the anti-tumor actions of paclitaxel. In addition, several studies have reported a specific anti-tumor effect of fenofibrate, thus increasing the interest in this drug as a potential treatment of CIPN [41,42,43]. In addition, treatment with fenofibrate is generally safe and effective as it has been used since 1975 for the treatment of long-term (>2 years) dyslipidemia in patients with normal liver and kidney function [44]. Fenofibrate can cause rare side effects (nausea, muscle weakness and cramps) and good tolerance in patients and its use is associated with a slightly increased risk (<1.0%) for myopathy [18,45]. However, monitoring of plasma levels of low-density lipoprotein (LDL) cholesterol, high-density lipoprotein (HDL) cholesterol, creatinine kinase (CK) and creatinine phosphokinase (CPK) should be performed for patient follow-up.

## 4. Materials and Methods

### 4.1. Animals

Ethical statement: All animal experiments were performed approved by the Association for Assessment and Accreditation of Laboratory Animal Care (AALAC) at Virginia Commonwealth University (Richmond, VA, USA). Experimental protocols were specifically approved by the Institutional Animal Care and Use Committee at Virginia Commonwealth University (protocol number: AM10142). A total of 96 mice were used, with 24 used to assess bioavailability and tolerability of fenofibrate diets (*n* = 8/group) and 72 used to assess the effects of fenofibrate diets on paclitaxel-induced peripheral neuropathy (*n* = 12/group). The experiments were performed on C57BL/6J female and male mice (12 weeks old) purchased from The Jackson Laboratory (Bar Harbor, ME, USA). Animals were housed at 4 per cage with an enriched environment and maintained at a room temperature of 22 °C, in a 12-h light/dark cycle. Mice had ad libitum access to regular food or fenofibrate diet and water. All behavioral experiments were performed during the light cycle and with the observer unaware of the treatment of the animals. Animals were randomly assigned to groups and baseline (BL) tests were performed for each behavioral test. The behavioral and electrophysiological experiments were carried out in two stages. All efforts were made to reduce the number of animals used in this study and to ensure optimal conditions of well -being before, during and after each experiment. The sample size for behavioral experiments was based on previous studies. Sample size calculations for the electrophysiological, tissue levels and biochemical outcomes (nerve conductance, epidermal nerve fiber density, cytokine and chemokine levels and mRNA expression levels) were performed following initial studies to inform the ongoing study. Here, *n* = 12 was required to be adequately powered for electrophysiological and mRNA expression studies. Furthermore, *n* = 8 was required for to be adequately powered for tissue levels and biochemical outcomes (epidermal nerve fiber density and cytokine and chemokine level studies). All mice were observed daily for general well-being and their weight was measured every other day. Any subjects that showed behavioral disturbances unrelated to chemotherapy-induced pain were excluded from further behavioral testing. Animal studies are reported in compliance with the ARRIVE guidelines [46].

### 4.2. Drug and Treatments

Fenofibrate (F6020-100G) was purchased from Sigma-Aldrich (St. Louis, MO, USA). Fenofibrate diet with 0.2% or 0.4% fenofibrate was prepared by the company Envigo with regular food (global 18% protein chow diet; Envigo Teklad, Indianapolis, IN, USA). The fenofibrate diet was started 5 days before treatment with paclitaxel and continued for 3 weeks. The doses of fenofibrate (0.2% and 0.4%) were determined in accordance with the literature [47,48].

Paclitaxel was purchased from VCU Health Pharmacy (Athenex, NDC 70860-200-50, Richmond, VA, USA) and dissolved in a 1:1:18 mixture of 200 proof ethanol, kolliphor and distilled water (Sigma-Aldrich). Paclitaxel was administered at a dose of 8 mg/kg intraperitoneally every other day; four administrations completed one regimen. Control mice received 1:1:18 at a volume of 10 mL/kg, i.p., and followed the same injection regimen.

For in vitro studies, paclitaxel (Athenex, NDC 70.860-200-50, Richmond, VA, USA) was dissolved in dimethyl sulfoxide (DMSO) (Sigma-Aldrich, St. Louis, MO, USA), diluted with sterile phosphate-buffered saline (PBS) and added to the incubation medium to achieve the indicated drug concentrations. Fenofibric acid (90568-10MG, Sigma-Aldrich, St. Louis, MO, USA) was dissolved in DMSO and added to the incubation medium to achieve the indicated drug concentrations. The in vitro concentrations of fenofibric acid were determined from the Cmax plasma concentrations from previously reported patient samples [49,50,51]. Cells were treated for 24 h; the DMSO concentration used never exceeded 0.1% DMSO. All studies with light-sensitive drugs were performed in the dark.

### 4.3. Von Frey Filaments Test

Mechanical hypersensitivity was determined using the Von Frey filaments test [27]. Briefly, mice were individually placed in cages on a mesh floor (Bioseb-PVF, Bioseb, Chaville, France) 30 min prior to testing. Mechanical thresholds were measured by applying Von Frey filaments in incremental force from 0.07 to 3.6 g to the plantar surface of each hind paw with the intent to elicit an appropriate response (paw withdrawal). Each filament was placed vertically against the paw surface with sufficient force for (approximately 3 s). Stimulation of the same filament was applied 3× at 10-second intervals. In the absence of response to a specific filament, a thicker filament and stronger stimulus were selected.

### 4.4. Acetone Test

Cold hypersensitivity was assessed via the acetone test [27]. Briefly, mice were placed individually into a cage with mesh metal flooring (Bioseb-PVF, Bioseb, Chaville, France) for 30 min prior to testing. Then, 20 mL of acetone (Sigma-Aldrich, MO, USA) was applied onto the plantar surface of each hind paw using a pipette. Time spent licking or shaking the hind paw was recorded for 60 s.

### 4.5. Locomotor Activity

Locomotor performance was determined using the locomotor activity test. Fenofibrate-treated (0.2% and 0.4%) and vehicle-treated male and female C57BL/6J mice (*n* = 8/sex/group) were placed in a room to acclimatize for 30 min before experimental testing. Mice were individually placed in boxes (20 × 24 × 16.5 cm, Omnitech, Columbus, OH) where locomotor activity was recorded by MED-PC IV software in 10-min intervals for 30 min. The test was performed 2 h after the last injection of fenofibrate, choline-fenofibrate or vehicle (after 7 days of treatment). Locomotor activity scores were defined as the number of interruptions of the photobeam cells measured for total test period. Data were expressed as mean of the number of photocell interruptions.

### 4.6. Wheel Running Test

Mouse motivation was determined by distance traveled using the wheel running test as previously reported [19]. Briefly, the mice were placed in wheels with a diameter of 21.5 cm and a width of 5 cm. An electronic sensor recorded the number of complete rotations of the wheel during a 2-h session (10 a.m.–12 p.m.). The equipment for this test was built at the Commonwealth University of Virginia, Richmond, VA, USA. The distance traveled was determined by multiplying the number of rotations performed and the circumference of the wheel (0.68 cm). The animal had access to only one direction of rotation.

### 4.7. Measurement of Caudal Nerve Conduction

The electrophysiological studies were performed at 21 days post-paclitaxel injection. Mice were anesthetized using a constant stream of 2.5% isoflurane in oxygen using a face mask and vaporizer (VetEquip Inc, Pleasanton, CA, USA). The dorsal caudal nerve conduction was recorded in tail with needle electrodes and the electrophysiological recording device PowerLab/26T (ADInstruments, Colorado Springs, USA). The recording needle electrodes were placed at the base of the tail at 0.5 cm. Stimulating needle electrodes were placed at the end of the tail at 5 cm. The ground electrode was placed between the two at 2.5 cm. The sensory nerve conduction was measured after stimulation with a supramaximal impulse of 0.1 ms and 5 Hz and intensities of 8 mA. The latency of the compound sensory action potential and the corresponding amplitudes were recorded.

### 4.8. Quantification of Intra-Epidermal Nerve Fibers (IENFs) by Immunohistochemistry

The staining procedure was based on a previously described method [27]. Briefly, skin of the hind paw was fixed in 4% paraformaldehyde at 4 °C overnight. The samples were embedded in optimal cutting temperature compound OCT and sectioned at 5 μm with a Cryostat. Sections were incubated in blocking solution (5% goat serum and 0.3% Triton X-100 in PBS) for 30 min at room temperature. Then, sections were incubated with the primary antibody anti-PGP9.5 (Fitzgerald—cat# 70R-30722, MA, USA) at 4 °C in a humidity chamber overnight (1:1000). After washes, sections were incubated with secondary antibody goat anti-rabbit IgG (H+L) conjugated with Alexa Fluor^®^ 594 (Life Technologies—cat# A11037, OR, USA) for 1 h and 30 min at room temperature (1:250). Finally, sections were examined using a Zeiss Axio Imager A1—Fluorescence microscope (Carl Zeiss, AG, Germany). The IENFs in each section were counted by one blinded examiner and the density of fibers is expressed as fibers/mm.

### 4.9. Electronic Microscopy of Sciatic Nerve

Sciatic nerves were collected and fixed in 2.5% glutaraldehyde in sodium cacodylate buffer at 4 °C overnight, rinsed and post-fixed in 1% osmium tetroxide in 100 mM cacodylate buffer, pH 7.3. Samples were then dehydrated and embedded in epoxy resin. Ultrathin longitudinal sections were collected on mesh copper grids and stained with uranyl acetate and lead citrate. Sections were then examined using a Jeol JEM-1230 transmission electron microscope at 100 keV. Ultra-thin sections were used for nerve morphological and mitochondria ultrastructure evaluation which was conducted by one blinded examiner.

### 4.10. Cytokines Analysis by Multiplex Assay

The mice were euthanized by decapitation. Dorsal root ganglion (DRG) and spinal cord tissues were collected and immediately frozen in liquid nitrogen and stored at −80 °C. DRG and spinal cord were homogenized and sonicated in 20 volumes of lysis buffer (25 mM Tris- HCl, 150 mM NaCl, 1 mM EDTA; pH 7.4) and supplemented with 50 mM NaF, 1 mM PMSF, protease and phosphatase inhibitor cocktails (50 μL/g of tissue and 10 μL/mL of lysis buffer, respectively). Lysates were sonicated and centrifuged at 15,000× *g* for 15 min at 4 °C. The resulting supernatants were collected for Qubit protein assay according to the manufacturer’s protocol. Supernatants were stored at −80 °C while waiting for the multiplex analysis. The level of cytokine expression was measured by the use of a Bio-Plex assay plate (Bio-Plex Pro Mouse Cytokine 23-plex Assay, Bio-Rad, CA, USA) according to the manufacturer’s protocol and the MAGPIX multiplex instrument with associated software (Bio-Rad, Hercules, CA, USA).

### 4.11. qRT-PCR

RNA isolation was performed using the Invitrogen™ PureLink™ RNA Mini Kit Ambion (ThermoFisher scientific, Waltham, MA, USA). The RNA concentration was measured using a QuBit 3.0 fluorimeter (ThermoFisher scientific, Waltham, MA, USA). In order to obtain cDNA, reverse transcription was performed on 20 ng/µL of RNA using the iScript™ cDNA Synthesis Kit (Bio-Rad, Hercules, CA, USA). Quantitative real-time PCR (qRT-PCR) was used to quantify PPAR-α (Mm00440939_m1 ppara, Thermo Fisher Scientific) mRNA expression. Expression of PPAR-α was normalized using reference gene: GAPDH (Glyceraldehyde-3-Phosphate Dehydrogenase) (Mm99999915_g1 Gapdh, Thermo Fisher Scientific). qRT-PCR was performed using a QuantStudio™ 3 Real-Time PCR System (Thermo Fisher Scientific, Waltham, MA, USA) and the qRT-PCR kit TaqMan™ Gene Expression Master Mix (Thermo Fisher Scientific, Waltham, MA, USA). A cycle threshold (Ct) value for each vial was determined using a threshold of 10^−1^. This value was chosen as it was clearly above the background fluorescence level. A melting curve analysis was performed which showed single product-specific melting temperatures. No primer dimers were generated during the 40 RT-PCR amplification cycles employed. All samples were quantified in triplicate for analysis. All values were standardized according to the reference genes.

### 4.12. UPLC-MS/MS Method for the Analysis of Fenofibrate and Fenofibric Acid

Mice were anesthetized (4% isoflurane), intracardiac blood was collected and then mice were euthanized by decapitation and brain, spinal cord and sciatic nerve were collected. Plasma and tissues were analyzed for fenofibrate and fenofibric acid (Cayman Chemical Company, Ann Arbor, Michigan, USA) using a modified previously published method [52]. The modified method was validated for precision and bias, recovery, matrix effects and specificity [53] in mouse plasma using the following quality control specimens: low (3 ng/mL fenofibrate and 30 ng/mL fenofibric acid), mid (30 ng/mL fenofibrate and 300 ng/mL fenofibric acid) and high (75 ng/mL fenofibrate and 750 ng/mL fenofibric acid).

### 4.13. Cell Culture

The 4T1 breast cancer cell line was obtained from the American Type Culture Collection (ATCC), Manassas, VA. Cells were maintained in Dulbecco’s modified Eagle’s medium (DMEM) supplemented with 10% (*v/v*) fetal bovine serum (FBS) (R&D Systems, S11150H, Minneapolis, MN, USA) and 1% (*v/v*) combination of 100 U/mL penicillin and 100 μg/mL streptomycin (Invitrogen, 15140-122, Carlsbad, CA, USA). Cells were cultured at 37 °C under humidified 5% CO2 conditions.

### 4.14. Cell Viability Assay

For cell viability assessed by Trypan blue exclusion, cells were plated in 6-well plates at a density of 50,000 cells per well and treated with fenofibric acid (20 or 40 nM), paclitaxel (50 nM) or the combination of fenofibric acid and paclitaxel for 24 h. Cells were trypsinized, stained with 0.4% Trypan blue (Sigma, T01282, St. Louis, MO, USA) and counted on the indicated days. Cellular growth curves were generated from the collected data.

### 4.15. Cell Growth Assay

The ovarian cancer cell lines SKOV-3 and CAOV-3 were obtained from the ATCC, Manassas, VA. SKOV-3 and CAOV-3 cells were cultured in RPMI1640 supplemented with 10% FBS. Cells were plated in 12-well plates (1 × 10^5^ cells/well). When they reached approximately 50% confluence, the cells were treated for 2 days with paclitaxel (PAC), fenofibric acid (FA) or their combination at concentrations detailed in legend of Figure 8. The attached cells were trypsinized and cell numbers/well were quantified with a Coulter counter.

### 4.16. Clonogenic Survival Assay

Cells were plated in 6-well plates at a density of 200 cells per well and treated with fenofibric acid (20 or 40 nM), paclitaxel (50 nM) or the combination of fenofibric acid + paclitaxel for 24 h. Drugs were removed after the 24-h period and cells were replenished with fresh medium and allowed to grow for ~14 days. Colonies were then washed with 1× phosphate-buffered saline (PBS, Life Technologies, Carlsbad, CA, USA), fixed with 100% methanol and stained with 0.1% crystal violet (Sigma, St. Louis, MO, USA). The number of colonies formed was counted visually.

### 4.17. Assessment of Apoptosis

The extent of apoptosis was monitored by Annexin V-FITC and propidium iodide staining. Cells were treated with fenofibric acid (20 or 40 nM), paclitaxel (50 nM) or the combination of fenofibric acid + paclitaxel for 24 h. On the day of analysis, cells were trypsinized, washed with 1X PBS and stained 48 h post-treatment according to the manufacturer’s protocol (Annexin V-FITC Apoptosis Detection Kit; BD Biosciences, 556547, San Jose, CA, USA). Fluorescence was measured by flow cytometry using BD FACSCanto II and BD FACSDiva software at the Flow Cytometry Core Facility at Virginia Commonwealth University. For all flow cytometry experiments, 10,000 cells per replicate were analyzed and three replicates for each condition were analyzed per independent experiment.

### 4.18. Statistical Analysis

All data are expressed as mean ± SEM (standard error of the mean). Normality and equal variance were verified by Shapiro–Wilk and Brown–Forsythe tests, respectively. Data were then compared using either a *t*-test or a one- or two-way analysis of variance (ANOVA) with repeated measures, followed by post-hoc Tukey’s comparison test. All statistical analyses were performed with GraphPad statistical software (GraphPad Software, Inc., La Jolla, CA, USA). Differences were considered significant when *p* was <0.05, <0.01 or <0.001.

## 5. Conclusions

In conclusion, our study shows, for the first time, the therapeutic potential of fenofibrate in the prevention of signs of paclitaxel-induced peripheral neuropathy. These effects are likely mediated by PPAR-α, resulting in the reduction in nerve fiber damage and neuroinflammation without compromising the anti-tumor effects of paclitaxel. Thus, fenofibrate, already widely used clinically for the treatment of dyslipidemia with a well-documented safety profile, could be the subject of therapeutic repurposing for the treatment of PIPN.

## Data Availability

Not applicable.
